# Correlation and diagnostic significance of CD4 T cell subsets and NLRP3 inflammasome in ulcerative colitis: the role of the NLRP3/T-bet/GATA3 axis

**DOI:** 10.1186/s12876-025-03603-w

**Published:** 2025-01-21

**Authors:** Yingnan Hu, Jingyi Tang, Dian Yu, Shuo Su, Jintao Fang, Linying Xia, Wenjun Xu, Weihan Zhu, Ninping Song, Fengyong Wang, Dechang Diao, Wei Zhang

**Affiliations:** 1 Present Address: The Second Clinical Medical College of Zhejiang Chinese Medical University, Zhejiang Chinese Medical University, Hangzhou, China; 2 Department of Spleen and Stomach Diseases, Qujiang District Hospital of Traditional Chinese Medicine, Quzhou, China; 3 Department of Orthopaedics, Zhoushan Hospital of Traditional Chinese Medicine Affiliated to Zhejiang Chinese Medical University, Zhoushan, China; 4 Present Address: Department of Colorectal Surgery, The Sixth Affiliated Hospital of Sun Yat-Sen University, Guangzhou, China; 5 Department of Gastrointestinal Surgery, The Second Affiliated Hospital of Zhejiang Chinese Medical University, No. 318, Chaowang Road, Gongshu District, Hangzhou City, Zhejiang Province CN310005 People’s Republic of China

**Keywords:** Ulcerative colitis, CD4 T cell, NLRP3 inflammasome, Correlation, Diagnostic value

## Abstract

**Background and aim:**

Ulcerative colitis (UC) is characterized by complex immunological interactions involving CD4 T cell subsets and the NLRP3 inflammasome, which influence inflammatory responses. This investigation focused on delineating the activation profiles of these components and their correlation with disease severity and activity, assessing their diagnostic implications in UC.

**Methods:**

We conducted immunohistochemistry and ELISA assays to measure markers expression of CD4 T cell subsets and the NLRP3 inflammasome in UC patients versus controls. Findings were validated using correlation analysis, molecular docking and ROC curves.

**Results:**

UC patients displayed increased Th1 (T-bet, TNF-α), Th2 (GATA3, IL-6), and Th17 (RORγt, IL-17, IL-22, IL-23) markers versus controls. Additionally, Th1 and Th2 cytokines (IL-2 and IL-4) were significantly elevated in severe UC, while Treg markers (FOXP3, IL-10, TGF-β1) were elevated only in mild-to-moderate UC. Enhanced NLRP3 inflammasome activation, indicated by elevated NLRP3, Caspase-1, and IL-1β levels. These molecular patterns, confirmed through correlation analysis and molecular docking, underscored strong correlations among NLRP3, T-bet, and GATA3, supporting the proposed NLRP3/T-bet/GATA3 axis. This axis, along with other biomarkers, showed strong associations with UC severity, Mayo score, UCEIS, demonstrated relatively high diagnostic value.

**Conclusion:**

The NLRP3/T-bet/GATA3 axis provides a referable strategy for multi-targeted combined treatment of UC and may serve as potential biomarkers for enhancing diagnostic accuracy and guiding therapy.

**Supplementary Information:**

The online version contains supplementary material available at 10.1186/s12876-025-03603-w.

## Introduction

Ulcerative colitis (UC) is a chronic inflammatory bowel disease (IBD) that primarily affects the mucosal lining of the colon and rectum, presenting with long-term recurrent abdominal pain, diarrhea, and bloody mucus-stained stools, while lacking any curative treatments at present, profoundly impacting patients' quality of life and imposing substantial socioeconomic burden [1, 2]. The pathogenesis of UC encompasses complex interactions among immune dysregulation, environmental factors, genetic predisposition, and intestinal flora [3]. CD4 T cell subsets exerts a prominent role in the immune inflammatory response of UC within the intestinal mucosa [4, 5], with their functional impairment and cytokine production imbalance being crucial factors in UC's complex pathophysiology [6]. Additionally, NLRP3 inflammasome was identified as a pivotal element in the inflammatory cascade of UC, which is a multi-protein complex that activates caspase-1 to convert pro-inflammatory cytokines into their active forms, thereby leading to the perpetuation of mucosal inflammation [7, 8]. Targeting NLRP3 inflammasome inhibition has demonstrated promise as a therapeutic strategy for UC, reducing IL-1β and IL-18 production in peripheral blood and potentially preventing intestinal fibrosis [9, 10]. However, a phase 1b trial evaluating selnoflast for moderate to severe UC confirmed its safety and tolerability but yielded mixed results regarding efficacy [11, 12]. Although some patients exhibited reduced inflammation, overall treatment responses were comparable to existing therapies, indicating that targeting solely the NLRP3 inflammasome may be inadequate. Therefore, a multi-target therapeutic approach addressing both the NLRP3 inflammasome and associated immune cell populations could be more effective for managing UC.

The interaction between the NLRP3 inflammasome and CD4 T cell subsets warrants attention. Overactivation of the NLRP3 inflammasome results in increased IL-1β production, disrupting tight junctions in the colonic epithelium, exacerbating UC onset, and impairing CD4 T cell function and differentiation [13–16]. Our previous studies on UC in BALB/c mice indicated that anti-UC effects involve balancing Th2/Th1 and Treg/Th17 subsets while inhibiting excessive NLRP3 inflammasome activation [17]. Further exploration of this interaction in clinical practice may offer new strategies for diagnosing and treating UC.

This clinical investigation aimed to elucidate the correlation between indices of CD4 T cell subsets and the expression of NLRP3 inflammasome components in UC patients. By identifying these associations, the study sought to uncover potential biomarkers associated with disease severity, activity, therapeutic responsiveness, and diagnostic value of UC, thereby focusing on the NLRP3/T-bet/GATA3 axis to deepen understanding of its role in UC and potentially refine treatment strategies.

## Methods

### Study participants

42 cases with UC and 30 patients with colon polyps were admitted to the main unit of the Second Affiliated Hospital of Zhejiang Chinese Medicine University from September 2021 to September 2023. Inclusion criteria were [3]: (I) Comprehensive diagnosis of UC and colon polyps based on clinical, laboratory, endoscopic, and histopathological assessments; (II) UC severity categorized by Truelove and Witts’s criteria, lesion extent assessed by the Montreal classification, and disease activity evaluated using the Mayo and UCEIS score; UC patients were required to be in the active phase with a Mayo score ≥ 3 and UCEIS score ≥ 1; (III) Age 18–90 years, male or female; (IV) Complete clinical data and laboratory information. The exclusion criteria were: (I) Age < 18 or > 90 years; (II) Malignant tumors; (II) Mental illness; (IV) Pregnant and lactating female; (V) Colon polyps exclude inflammatory colon polyps.

### Data collection and sample preparation

Clinical and laboratory data were collected, including disease diagnosis, pathology reports, endoscopy results, patient admission information, routine peripheral blood tests, etc. Endoscopic biopsy specimens: mucosal lesions in UC patients and normal mucosa from polyp patients (≥ 10 cm from polyps) (Supplementary Fig. S1). Peripheral venous blood serum was collected, allowed to coagulate at room temperature for 30–60 min, centrifuged at 2000–3000 RPM for 5–10 min, and stored at -80 °C.

Importantly, the disease severity and activity of current UC patients were carefully assessed during specimen and serum sample collection, ensuring consistency between the experimental analyses and the actual disease conditions while minimizing potential confounding factors such as medications, comorbid conditions, or diet that could influence UC severity and activity.

### Immumohistochemical staining in tissues

Immunohistochemical (IHC) detection was performed to assess the expression of CD4 T cell transcription proteins (T-bet, GATA3, RORγt, FOXP3) and NLRP3 inflammasome key proteins (NLRP3-Protein (P), Caspase-1, and IL-1β-Protein (P)). Colon tissue specimens were placed in a 60 °C oven for 2 h to dry, then dewaxed in water, and washed with distilled water for 2 min. The specimens were then subjected to high-pressure heat fixation, and 3% H_2_O_2_ solution was used to block peroxidases for 10 min. Fresh colonic mucosal tissue samples were fixed for over 24 h, dehydrated through sequential gradients of alcohol and xylene, and then infiltrated with melted paraffin at 65 °C. The infiltrated tissues were embedded, trimmed, and sectioned to a thickness of 4 µm. Sections were flattened in water at 40 °C, picked up with glass slides, and baked in an oven at 60 °C before being stored at room temperature. For immunohistochemical analysis, paraffin sections from all study subjects were dewaxed in water and washed with distilled water for 2 min. The specimens were then subjected to high-pressure heat fixation, and 3% H2O2 solution was used to block peroxidases for 10 min. After washing with PBS for 5 min × 3 times, the detection reagents were applied, and the primary antibodies were added with the following specific dilution ratios: T-bet (1:100) and GATA3 (1:1000) from Cell Signaling Technology, Danvers, MA, USA; RORγt (1:100, Novusbio, Beijing, China), FOXP3 (1:200, Cohesionbio, Shanghai, China); NLRP3-P (1:1000), Caspase-1 (1:1000), and IL-1β-P (1:500) from Selleck Chemicals, Wuhan, China. The specimens were incubated at 37 °C for 60 min. After washing with PBS for 5 min three times, the secondary antibodies were added and incubated at 37 °C for 60 min. Subsequently, the sections were developed with DAB to visualize positive signals as brown-yellow and counterstained with hematoxylin to color the nuclei blue. The sections were then dehydrated through a graded alcohol series, cleared, and mounted. Five distinct, non-overlapping regions were randomly selected from each patient's tissue section for analysis using Image-Pro Plus software (Version 6.0, Media Cybernetics Inc., USA). The selection of these regions aimed to ensure the representativeness of the immunohistochemical (IHC) data and minimize potential biases. For each selected region, the integrated optical density (IOD) of the target protein was measured. The average optical density (AOD), calculated as AOD = IOD/area, was used to quantify the intensity of the positive staining signals in each region. AOD represents the strength of the positive staining signal, with higher AOD values indicating a higher level of target protein expression within the selected region. The mean AOD value of the five regions was then computed and used as the representative AOD value for each tissue section in subsequent statistical analyses.

### Enzyme-linked immunosorbent assay (ELISA)

ELISA was used to measure factors associated with CD4 T cell subsets (TNF-α, IFN-γ, IL-2, IL-4, IL-6, IL-10, IL-17, IL-22, IL-23, TGF-β1) and NLRP3 inflammasome (NLRP3-Factor (F), IL-18 and IL-1β-Factor (F)). NLRP3-F ELISA kits were from CUSABIO Biotech (Cusabio, Wuhan, Hubei, China), while the other kits were from Lianke Biotech (Lianke Bio, Hangzhou, Zhejiang, China). Serum samples and ELISA reagents were processed according to the manufacturer's instructions. Absorbance was measured at 450 nm using an Automatic Microplate Reader (BioTek Epoch 2, BIOTEK, USA) and standard curves were used to quantify factor levels.

### Molecular docking

We employed AutoDockTools 1.5.6 and PyMOL software for molecular docking and visualization to investigate the potential regulatory effects of targeting NLRP3 on the functions of T-bet and GATA3 in UC mechanisms. The NLRP3 inhibitor selnoflast was selected as a ligand from the PubChem database, while T-bet and GATA3 proteins were sourced as receptors from the PDB database [11]. The binding energy threshold of ≤ -5.0 kcal·mol^-1^ indicates a strong affinity between the ligand and receptors.

### Statistical analysis

Statistical analysis was performed using SPSS (version 25.0, IBM, Armonk, USA), and date visualization was accomplished with GraphPad Prism (version 8.0.1, GraphPad Software, San Diego, USA). Data with normal distribution and homogeneous variance were reported as mean ± standard error. Comparisons across multiple groups were made using one-way ANOVA with Tukey's post hoc test. For non-normally distributed data or data with heterogeneous variances, results were presented as median [interquartile range, M (IQR)]. Non-parametric analysis (Kruskal–Wallis test) was employed for multi-group comparisons and Games-Howell test was used for further pairwise group comparisons. P values < 0.05 were considered statistically significant.

Spearman's correlation analysis was employed to explore the associations between CD4 T cell subsets and NLRP3 inflammasome indicators, as well as with the severity of UC, Mayo scores and UCEIS. Correlation heatmaps were generated with the OmicStudio tool (https://www.Omicstudio.cn). Furthermore, the diagnostic value of above these markers was evaluated using receiver operating characteristic (ROC) curves, with the Area Under The Curve (AUC) reported in Supplementary Table S1.

## Results

### Baseline characteristics of UC patients and colon polyps controls

**Fig. 1:**
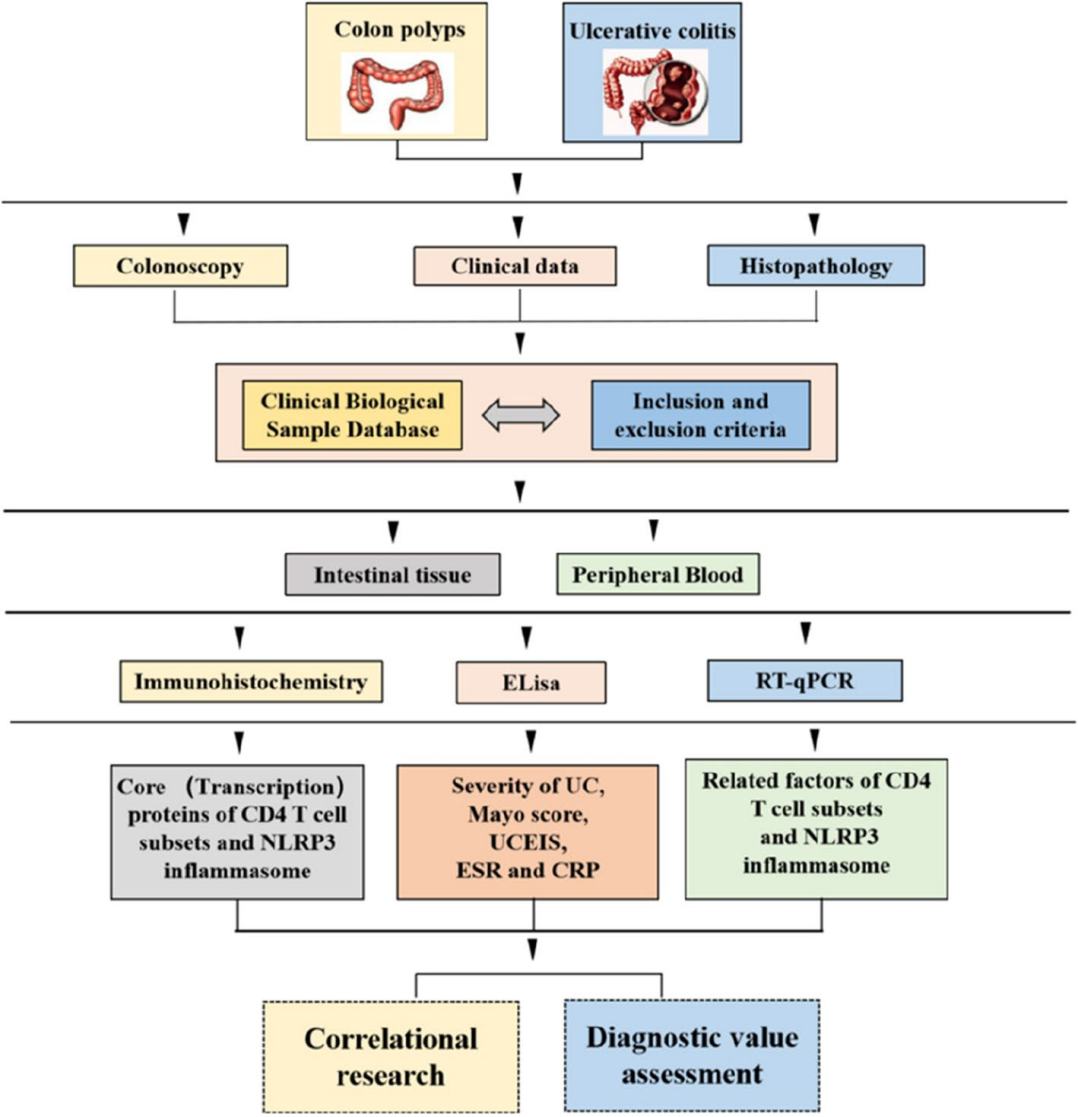
Research flowchart. The graph created by Figdraw (https://www.figdraw.com)

A total of 42 UC patients with active disease and 30 patients with colon polyps controls (CON) were included. UC patients were categorized based on Truelove and Witts' criteria into 22 with mild to moderate UC (UC-LM) and 20 with severe UC (UC-S). Baseline data, encompassing age and gender, the history of smoking and alcohol consumption, liver function markers (ALT, AST, AST/ALT ratio), and tumor biomarkers (AFP, CEA, CA199, CA125, and CA724), exhibited no statistically significant disparities between the CON and the UC groups among UC subgroups (*P* > 0.05). UC patients had higher BMI and HGB levels but lower CRP and WBC levels compared to CON group (*P* < 0.0001). The medication history in this study included aminosalicylates, corticosteroids, immunosuppressive drugs (ISD), and biologics. None of the colon polyp patients had a history of such usage. In the UC-LM and UC-S groups, only aminosalicylates showed statistical significance (*P* = 0.006), whereas glucocorticoids, immunosuppressive drugs (ISD), and biologics did not (*P* > 0.05). Detailed demographics and clinical characteristics are provided in Table 1 and the research flowchart in Fig. 1.

**Table 1:** Baseline characteristics of Ulcerative colitis (UC) patients and Colon polyp controls (CON)

Characteristics	CON group (*n* = 30)	UC group (*n* = 42)	UC-LM group (*n* = 22)	UC-S group (*n* = 20)	*P* value^a^	*P* value^b^	*P* value^c^	*P* value^d^
Age (years)	60 (53.50–65)	66 (45–74)	64.5 (42.5–69.5)	66.5 (45–74)	0.525	0.254	0.286	0.456
Sex					0.750	0.683	0.792	0.918
Male	16 (63.3%)	24 (57.1%)	13 (59.1%)	11 (55.0%)				
Female	14 (36.7%)	18 (42.9%)	9 (40.9%)	9 (45.0%)				
BMI (kg/m^2^)	23.21 ± 1.92	20.85 ± 1.88	21.82 ± 1.48	19.78 ± 1.70	< 0.0001	0.0068	0.0002	< 0.0001
Disease duration					< 0.0001	< 0.0001	0.002	< 0.0001
< 2 years	26 (86.7%)	17 (40.5%)	14 (63.6%)	3 (15%)				
2–5 years	3 (23.3%)	7 (16.7%)	5 (22.7%)	2 (10%)				
> 5 years	1 (3.3%)	18 (42.9%)	3 (13.6%)	15 (75%)				
Smoking history	7 (23.33%)	8 (19.05%)	3 (13.6%)	5 (25%)	0.661	0.385	0.355	0.606
Alcohol consumption history	9 (30%)	7 (16.67%)	3 (13.6%)	4 (20%)	0.183	0.171	0.585	0.365
Montreal classification					—	—	0.002	—
E1	—	4 (9.5%)	4 (18.18%)	0 (0%)				
E2	—	10 (23.8%)	8 (36.36%)	2 (10%)				
E3	—	28 (66.7%)	10 (45.46%)	18 (90%)				
WBC (× 10^9^/L)	5.85 ± 1.54	9.45 ± 3.77	8.20 ± 3.05	10.83 ± 4.06	< 0.0001	0.0007	0.0219	0.236
HGB (g/L)	131.40 ± 16.83	109.40 ± 16.39	120.20 ± 9.43	97.60 ± 14.16	< 0.0001	0.0068	< 0.0001	< 0.0001
CRP (mg/L)	2.05 (0.10–4.33)	18.69 (7.50–35.58)	8.79 (1.45–36.9)	23.95 (15.05–34.41)	< 0.0001	0.006	0.018	< 0.0001
ESR (mm/60 min)	—	23.00 (8.00–39.25)	10.50 (6.75–24.25)	31.50 (22.25–40.75)	—	—	0.002	—
ALB (g/L)	40.45 ± 3.64	35.34 ± 5.49	36.61 ± 4.78	33.94 ± 5.99	< 0.0001	0.0018	0.1163	< 0.0001
ALT (U/L)	17 (13.75–25.50)	18 (11–27)	23.50 (14–30.75)	14 (10–31)	0.899	0.286	0.136	0.242
AST (U/L)	22 (18–24)	22 (19–27)	24.5 (19.75–33)	21 (17–24.5)	0.456	0.127	0.122	0.195
AST/ALT	1.1 (0.93–1.43)	1.40 (0.90,1.70)	1.3 (0.90–1.50)	1.4 (0.90–1.90)	0.375	0.603	0.378	0.524
AFP (ng/mL)	2.8 (1.80–3.50)	2.55 (1.98–3.70)	2.50 (1.95–3.65)	2.70 (1.95–5.55)	0.675	0.959	0.628	0.791
CEA (ng/mL)	2.60 (1.80–3.90)	2.40 (1.70–3.93)	2.20 (1.70–4.50)	2.60 (1.70–3.45)	0.627	0.467	0.769	0.81
CA199 (ng/mL)	5.30 (3.20–11.70)	7.85 (2.58–16.58)	4.80 (2.15–12.70)	12.40 (3.75–28.50)	0.699	0.506	0.102	0.188
CA125 (ng/mL)	8.20 (7.33–12.25)	12.20 (6.60–22.13)	10.80 (6.50–12.85)	15.90 (7.60–34.80)	0.069	0.392	0.134	0.064
CA724 (ng/mL)	2.20 (0.90–3.20)	1.60 (0.80–5.53)	1.30 (0.88–7.85)	1.80 (0.20–5.53)	0.861	0.846	0.796	0.982
Treatment					—	—	0.6857	—
Aminosalicylates	—	22	11	2	—	—	0.006	—
Corticosteroids	—	16	9	6	—	—	0.467	—
ISD	—	3	2	6	—	—	0.089	—
Biologics	—	10	7	11	—	—	0.134	—

*UC* ulcerative colitis, *UC-LM* mild to moderate ulcerative colitis, *UC-S* severe UC, *CON* colon polyp controls, *BMI* body mass index, *WBC* white blood cells, *HGB* hemoglobin, *CRP* C-reactive protein, *ESR* erythrocyte sedimentation rate, *ALB* albumin, *ALT* Alanine Aminotransferase, *AST* Aspartate Aminotransferase, *AFP* Alpha-Fetoprotein, *CEA* Carcinoembryonic Antigen, *CA199* Cancer Antigen 199, *CA125* Cancer Antigen 125, *CA724* Cancer Antigen 724, *ISD* Immunosuppressive drug

^a^comparing CON group and UC group

^b^comparing CON group and UC-LM group

^c^comparing UC-LM group and UC-S group

^d^comparing CON group, UC-LM group and UC-S group

### Distribution of CD4 T cell subsets in intestinal tissue

**Fig. 2:**
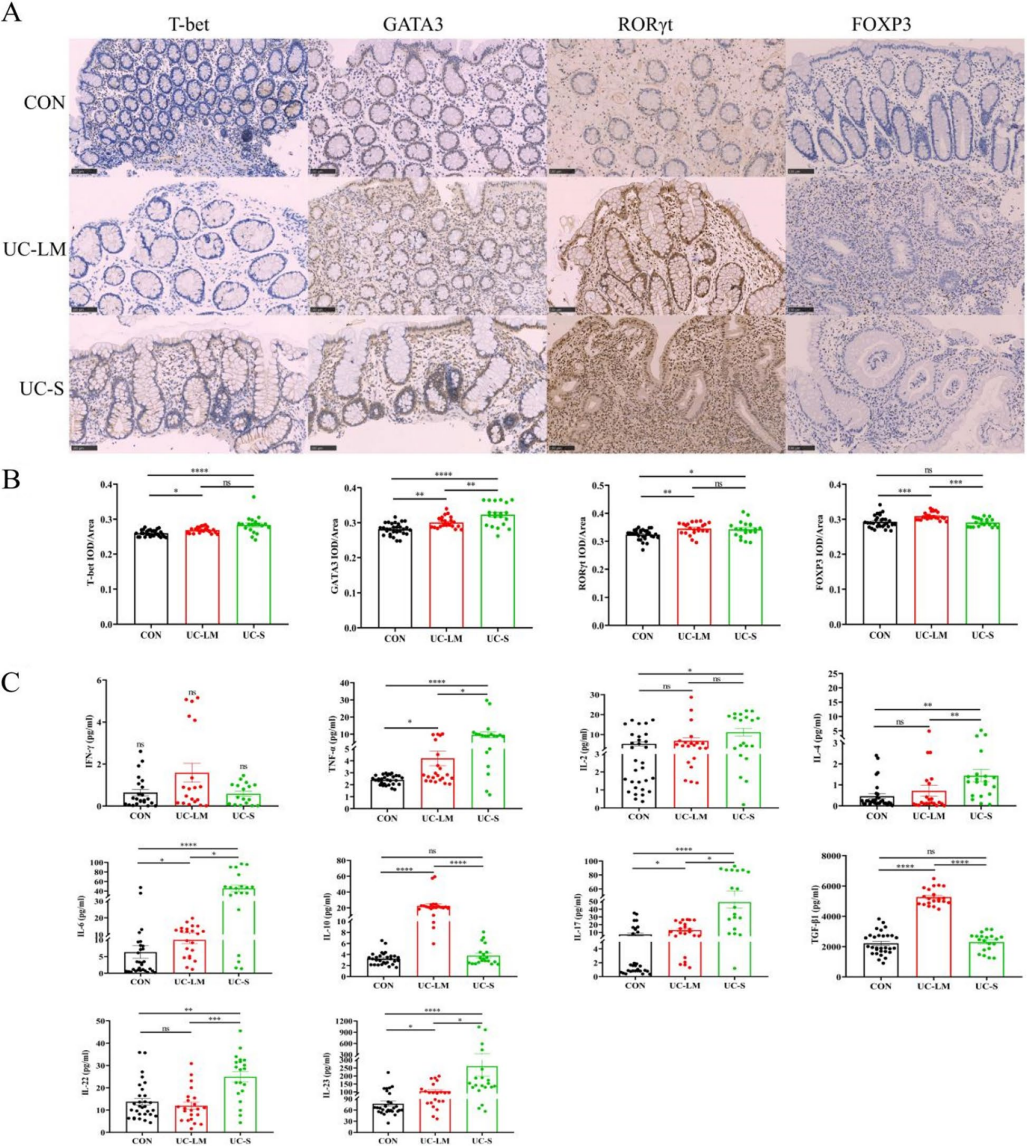
The distribution of CD4 T cell subsets (Th1, Th2, Th17, and Tregs) in intestinal tissues was detected by immunohistochemical staining for T-Bet, GATA3, RORγt, and FOXP3, and serum levels of CD4 T cell subset-related cytokines were measured by ELISA in the CON, UC-LM, and UC-S groups. **A** Representative immunohistochemical staining of T-bet, GATA3, RORγt and FOXP3. **B** Immunohistochemical results of T-bet, GATA3, RORγt and FOXP3. **C** TNF-α, IFN-γ, IL-2, IL-4, IL-6, IL-10, IL-17, IL-22, IL-23, and TGF-β1 were analyzed by ELISA. Data were expressed as mean ± SEM (*n* = 20–30): *P < 0.05, **P < 0.01, ***P < 0.001, ****P < 0.0001

Immunohistochemical staining of tissue sections from the CON, UC-LM, and UC-S groups was performed to evaluate CD4 T cell subset (Th1, Th2, Th17 and Tregs) distribution using antibodies against T-bet, GATA3, RORγt, and FOXP3 (Fig. 2A). Increased expression of these proteins was predominantly observed in the mucosal and submucosal layers of active UC. Compared to the CON group, the UC-LM group showed significantly higher levels of T-bet (*P* < 0.05), RORγt (*P* < 0.01), and GATA3 and FOXP3 (*P* < 0.001) (Fig. 2B). The UC-S group had even greater levels of T-bet and GATA3 (*P* < 0.0001) and RORγt (*P* < 0.05), with no significant difference in FOXP3 (*P* > 0.05). Compared to UC-LM, the UC-S group had increased GATA3 (*P* < 0.01) and decreased FOXP3 (*P* < 0.001), while T-bet and RORγt levels remained similar (*P* > 0.05).

### Expression of CD4 T cell subsets-related cytokines in peripheral serum

The expression levels of CD4 T cell subsets-related cytokines (TNF-α, IFN-γ, IL-2, IL-4, IL-6, IL-10, IL-17, IL-22, IL-23, TGF-β1) were detected by ELISA (Fig. 2C). Compared to the CON group, the UC-LM group exhibited remarkably increased expression of TNF-α, IL-6, IL-23 and IL-17 (*P* < 0.05), IL-10 and TGF-β1 (*P* < 0.0001), with no significant differences in IFN-γ, IL-2, IL-4, and IL-22 (*P* > 0.05). In UC-S group, TNF-α, IL-6, IL-17, and IL-23 (*P* < 0.0001), IL-2 (*P* < 0.05), IL-4 and IL-22 (*P* < 0.01) were significantly elevated, while IFN-γ, IL-10, and TGF-β1 showed no significant changes (*P* > 0.05). Compared to the UC-LM group, the UC-S group had increased levels of TNF-α, IL-6, IL-17, and IL-23 (*P* < 0.05), IL-4 (*P* < 0.01), IL-22 (*P* < 0.001), and along with decreased IL-10 and TGF-β1 (*P* < 0.0001), with no significant differences in IFN-γ and IL-2 (*P* > 0.05).

### Distribution of NLRP3 inflammasome in intestinal tissue

**Fig. 3:**
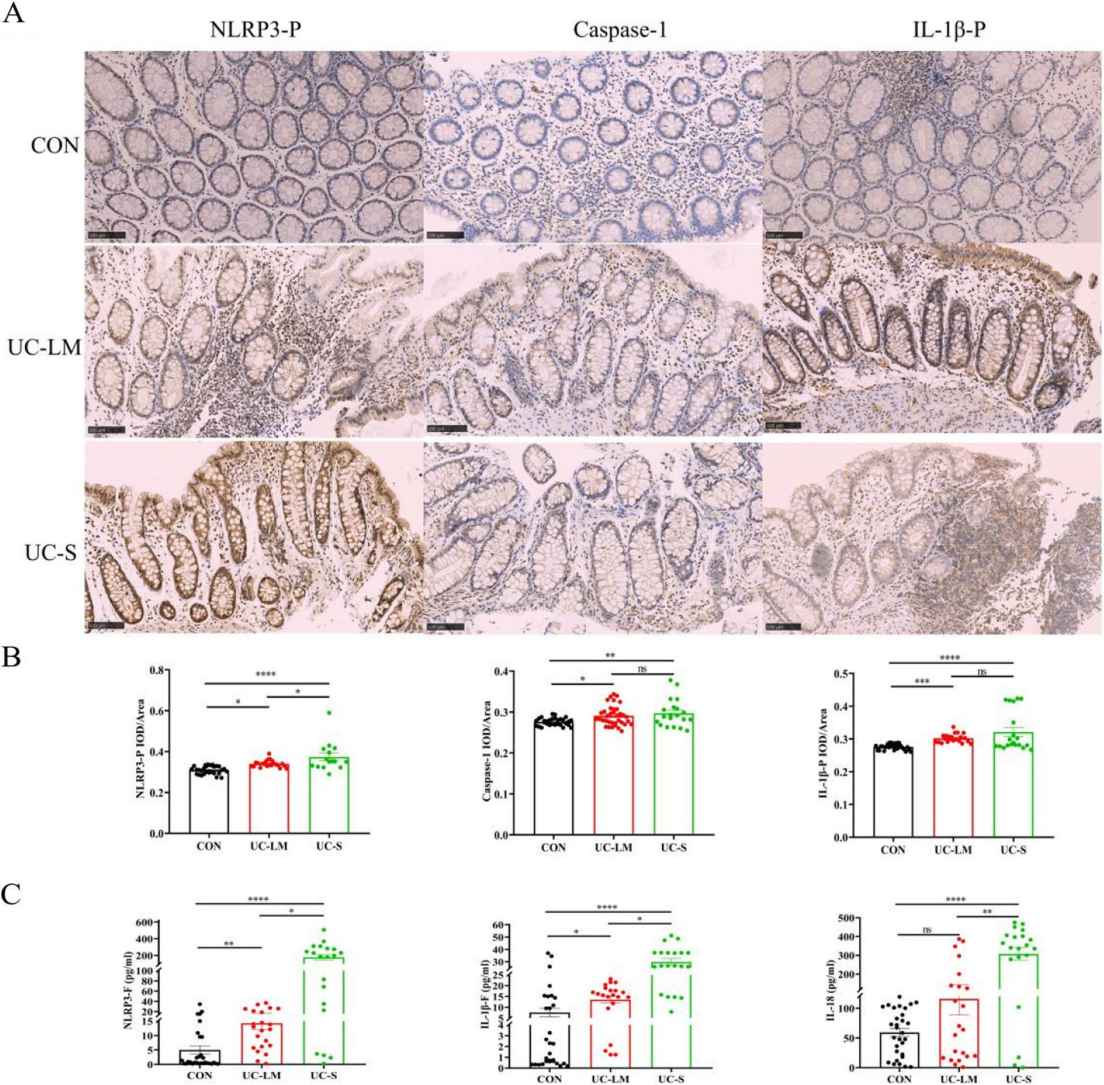
The distribution of the NLRP3 inflammasome in intestinal tissues was detected by immunohistochemical staining for NLRP3-P, Caspase-1, and IL-1β-P, and serum levels of NLRP3-F, IL-1β-F, and IL-18 were measured by ELISA in the CON, UC-LM, and UC-S groups. **A** Representative immunohistochemical staining of NLRP3-P, Caspase-1 and IL-1β-P. **B** Immunohistochemical results of NLRP3-P, Caspase-1 and IL-1β-P. **C** Expression of NLRP3 inflammasome-related factors in peripheral serum and intestinal tissues. a NLRP3-F, IL-1β-F and IL-18 were analyzed by ELISA; Data were expressed as mean ± SEM (*n* = 20–30): *P < 0.05, **P < 0.01, ***P < 0.001, ****P < 0.0001

Immunohistochemical staining was used to assess the distribution of NLRP3 inflammasome components (NLRP3-P, Caspase-1, IL-1β-P) (Fig. 3A). Elevated expression of these proteins was primarily observed in the mucosal and submucosal layers of active UC, particularly at sites of inflammatory aggregation. Compared to the CON group, the UC-LM group exhibited a significant elevation in the levels of NLRP3-P (*P* < 0.05), Caspase-1 (*P* < 0.05), and IL-1β-P (*P* < 0.001) (Fig. 3B). The UC-S group also showed markedly increased levels of NLRP3-P and IL-1β-P (*P* < 0.0001), and Caspase-1 (*P* < 0.01) compared to the CON group. When comparing UC-S to UC-LM, higher levels of NLRP3-P (*P* < 0.05), Caspase-1 and IL-1β-P (*P* > 0.05) were observed.

### Expression of NLRP3 inflammasome-related factors in peripheral serum

The expression levels of NLRP3 inflammasome-related factors (NLRP3-F, IL-1β-F, and IL-18) in peripheral serum were measured by ELISA (Fig. 3C). Compared to the CON group, the UC-LM group showed a significant increase in NLRP3-F and IL-1β-F (*P* < 0.01), with no significant difference in IL-18 (*P* > 0.05). The UC-S group exhibited a marked increase in NLRP3-F, IL-1β-F, and IL-18 (*P* < 0.0001). More details can be found in Supplementary Table S5.

### Correlation between the indicators of CD4 T cell subsets and NLRP3 inflammasome

**Fig. 4:**
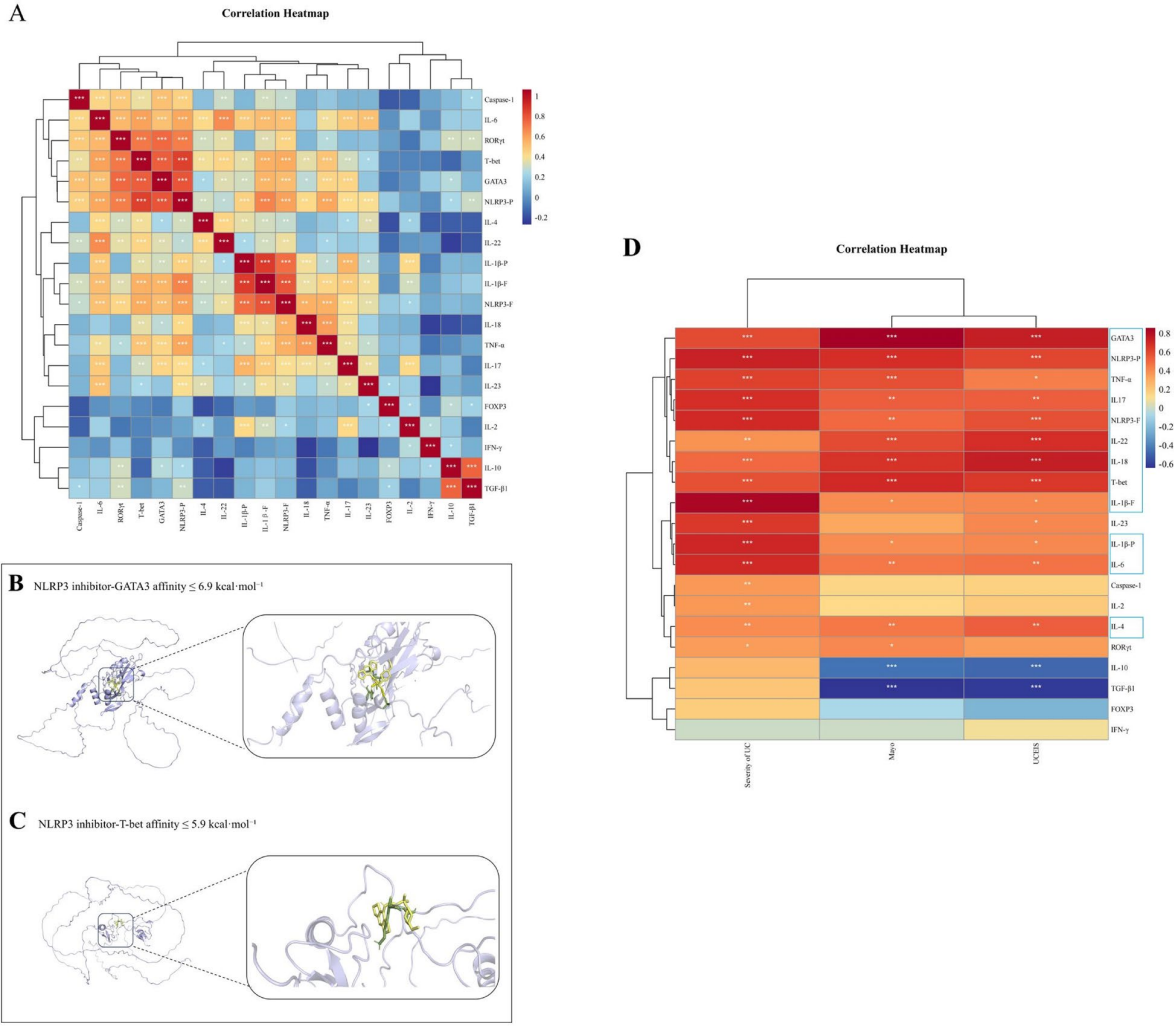
To explore the correlation between the indicators of both CD4 T cell subsets and NLRP3 inflammasome, as well as the correlations between the indicators of both CD4 T cell subsets and NLRP3 inflammasome with the severity of UC, Mayo score, and UCEIS. **A** CD4 T cell subsets and NLRP3 inflammasome-related indicators were shown with correlation heatmap. -P suffix indicated a protein, while the -F suffix indicated a factor. **B** Docking mode between NLRP3 inhibitor selnoflast and T-bet. **C **Docking mode between NLRP3 inhibitor selnoflast and GATA3. **D** CD4 T cell subsets and NLRP3 inflammasome-related core indicators were shown with Correlation Heatmap. -P suffix indicated a protein, while the -F suffix indicated a factor. Data were presented as mean ± SEM (*n* = 20-30): *P < 0.05, **P < 0.01, ***P < 0.001, ****P < 0.0001

To investigate the correlation between the indicators of CD4 T cell subsets and the NLRP3 inflammasome, correlation heatmaps were employed to visualize the results. (Supplementary Table S2, Fig. 4A). Among the 20 indicators analyzed, NLRP3-P showed the strongest correlation, being significantly related to 17 indicators. NLRP3-F and IL-1β-F were each correlated with 16 indicators, while GATA3 and T-bet were correlated with 15 indicators each (*P* < 0.05). Given the correlation degree, targeting the NLRP3/T-bet/GATA3 axis may provide a potential pathway for UC treatment. We employed molecular docking to explore how targeting NLRP3 affects GATA3 and T-bet (Fig. 4B-C), with the clinical trial mentioning the NLRP3 inhibitor selnoflast as a selection [11], which showed binding affinities of ≤ -5.9 kcal·mol⁻^1^ for NLRP3 inhibitor-T-bet and ≤ -6.9 kcal·mol⁻^1^ for NLRP3 inhibitor-GATA3, indicating good binding affinity and further supporting our hypothesis.

### Correlations between the indicators of both CD4 T cell subsets and NLRP3 inflammasome with the severity of UC, Mayo score, and UCEIS

To identify biomarkers reflecting the severity and activity of UC, we assessed the correlation between CD4 T cell subsets and NLRP3 inflammasome markers with the severity of UC, Mayo score, and UCEIS (Fig. 4D). 12 indicators showed a significant positive correlation with UC severity, Mayo score, and UCEIS (*P* < 0.05), including Th1 (T-bet, TNF-α), Th2 (GATA-3, IL-4, IL-6), Th17 (IL-17, IL-22), and NLRP3 inflammasome markers (NLRP3-P, NLRP3-F, IL-1β-F, IL-1β-P, IL-18). More details are available in Supplementary Table S3.

### Assessment of the diagnostic value of CD4 T cell subsets and NLRP3 inflammasome core indicators in the severity of UC patients

**Fig. 5:**
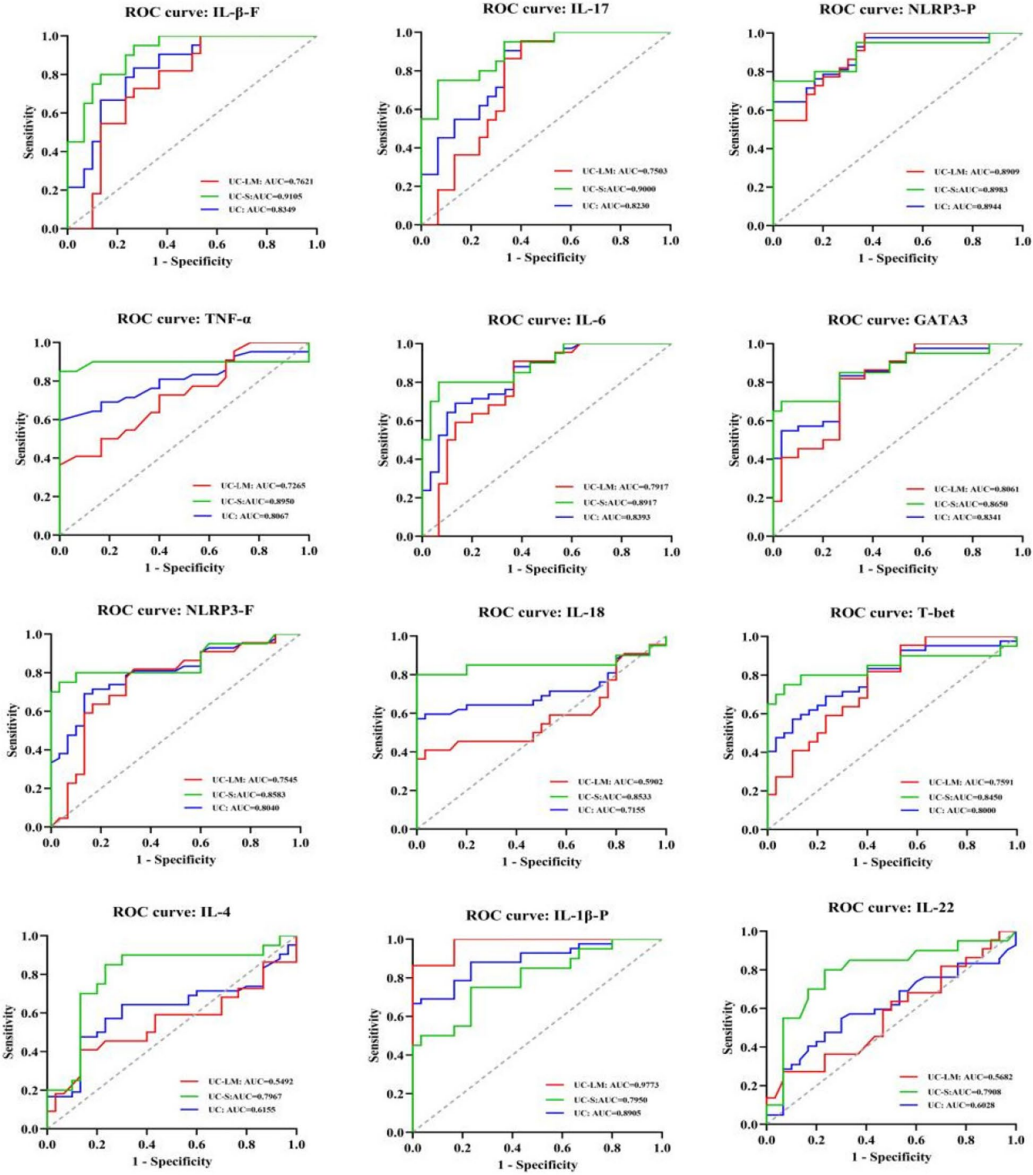
Assessment of the diagnostic value of CD4 T cell subsets and NLRP3 inflammasome core indicators in the severity of UC patients. ROC curves of IL-1β-F, IL-17, NLRP3-P, TNF-α, IL-6, GATA3, NLRP3-F, IL-18, T-bet, IL-4, IL-1β-P, IL-22 for assessing disease severity of UC patients. The blue line represented UC disease, the red line UC-LM, and the green line UC-S

We evaluated the diagnostic value of above 12 indicators (Fig. 5). For UC group, IL-4 and IL-22 had low diagnostic values, while other indicators showed moderate diagnostic values. Notably, NLRP3-P (AUC = 0.8944) and IL-1β-P (AUC = 0.8905) had higher AUC values. In UC-LM group, IL-1β-P (AUC = 0.9773) demonstrated high diagnostic value, whereas IL-4, IL-22, and IL-18 had lower values. For UC-S group, IL-1β-F (AUC = 0.9105) had high diagnostic values, with other indicators showing moderate values (AUC > 0.7900). The optimal biomarkers, categorized by proteins and factors, based on AUC values were: in UC group, NLRP3-P (AUC = 0.8944) and IL-6 (AUC = 0.8393); in UC-LM group, IL-1β-P (AUC = 0.9773) and IL-6 (AUC = 0.7917); in UC-S group, NLRP3-P (AUC = 0.8983) and IL-1β-F (AUC = 0.9105) (Supplementary Table S4).

## Discussion

In recent decades, the incidence and prevalence of UC have shown a continuous upward trend. As of 2023, the global UC patients population has risen to approximately 5 million cases, establishing UC as a common and intricate disease within the gastrointestinal system [3, 18]. This trend emphasizes the imperative requirement for comprehensive investigation into the mechanisms of UC, particularly its etiology, treatment, and prognosis, which remain pivotal focal points in research [19]. The pathogenesis of UC entails intricate interactions among diverse cellular subsets and cytokines [20–22]. Exogenous factors have the potential to inflict damage on the intestinal mucosa and disrupt immune tolerance, culminating in a dysregulated immune response to antigens derived from the gut microbiome. This aberrant response precipitates the uncontrolled activation of effector T cells, especially CD4 T cells, which subsequently undergo differentiation into subtypes such as Th1, Th2, Th17, and Tregs [19, 22].

The increase in Th2 cells as a primary immunological signature of UC is marked by elevated GATA3 and cytokines such as IL-4, IL-5, IL-6, and IL-13 [23–27]. Our research found that UC patients had significantly higher levels of GATA3, IL-4, and IL-6, which positively correlated with UC severity, Mayo score, and UCEIS, highlighting the critical role of Th2 cell activation in UC progression and clinical assessment. Th1 cells, through T-bet transcription factor regulation, produce secreted cytokines such as TNF-α, which are closely associated with the pathological mechanisms underlying UC [28, 29]. Moveover, the imbalance between Th1 and Th2 cells is a vital factor in exacerbating UC severity [30–32]. Biological agents targeting TNF-α directly intervene in UC inflammation are essential to management strategies, with TNF-α antagonists like adalimumab widely recommended [32, 33]. Our study revealed significantly elevated levels of T-bet and TNF-α in UC patients, particularly in severe cases, positively correlating with UC severity, Mayo score, and UCEIS (Fig. 2C, Supplementary Table S3), suggesting that increased T-bet may drive Th1 cell activation and TNF-α release. Remarkably, IFN-γ levels, a key secretory product of Th1 cells, showed no significant differences among the groups, marked contrast was observed with the increase in TNF-α levels, where increased Th2 cell activity and IL-4 secretion may inhibit IFN-γ production, indicating a need for targeted therapies focusing on cytokines like TNF-α in UC management. IL-2, secreted by Th1 cells, promotes T cell proliferation and immune tolerance, helping to prevent autoimmune diseases [34, 35]. In our study, elevated IL-2 levels in UC patients were positively correlated with disease severity, demonstrating that increased IL-2 exacerbates inflammation and immune dysregulation in UC, further supporting the notion that Th1 cells may be activated. Currently, the Tregs/Th17 immune balance is considered to be an important mediator leading to colonic mucosal ulcers and tissue damage in UC [36–38]. Tregs, by secreting TGF-β1 and IL-10, play an anti-inflammatory role essential for immune tolerance. Th17 cells, in turn, exacerbate inflammation through the secretion of IL-17, IL-22 and IL-23. UC patients typically show reduced Tregs and increased Th17 cells [38, 39]. This study found elevated Th17 levels in UC, indicated by significantly higher RORγt, IL-17, IL-22, and IL-23, all positively correlated with UC severity. Tregs exhibited a complex expression pattern, with only UC-LM patients showing increased FOXP3, TGF-β1, and IL-10 levels, the latter two negatively correlated with Mayo score and UCEIS. This reveals that early features of UC involve increased activity of Tregs and Th17 cells, which balance each other to reduce symptoms. As UC progresses, Treg secretion of anti-inflammatory factors decreases while Th17 pro-inflammatory cytokine levels, worsening the condition.

The NLRP3 inflammasome drives the release of IL-1β and IL-18, worsening inflammation and tissue damage, significantly promoting UC occurrence [40, 41]. NLRP3 gene polymorphisms influence IBD susceptibility, and NLRP3 core protein levels are elevated in active UC [11, 12]. These core protein levels in our study were increased to varying degrees and positively correlated with disease severity and activity indicators. Notably, IL-18 was significantly elevated only in severe UC, distinguishing it from the persistent elevation of IL-1β and revealing the complex NLRP3 inflammasome network in UC [42, 43]. In animal models of UC, a strong correlation has been observed between the NLRP3 inflammasome and CD4 T cell subsets [17, 44–46]. The interaction between the NLRP3 inflammasome and Th1/Th2 differentiation may involve the release of IL-1β and IL-18 [47, 48]. IL-1β enhances T-bet expression, promoting Th1 differentiation, while IL-18 also supports Th1 responses. GATA3, crucial for Th2 differentiation, may be regulated by NLRP3 through pathways like NF-κB or MAPK [49, 50]. Moreover, NLRP3 activation could affect the expression or post-translational modification of T-bet and GATA3, including phosphorylation and acetylation, modulating their roles in Th1/Th2 differentiation [50, 51]. These pathways help elucidate NLRP3’s role in immune responses in UC. However, the dynamics in clinical settings remain insufficiently explored. Through the analysis of their correlation, we found that NLRP3-P, T-bet and GATA3 had a high correlation (Fig. 4A, Supplementary Table S2), suggesting that NLRP3 inflammasome activity may regulate the expression and function of these transcription factors, thereby influencing Th1 and Th2 cell functions. Besides, Selnoflast, an NLRP3 inhibitor, has not met therapeutic expectations as a monotherapy in UC [11]. A combined targeting strategy may improve clinical outcomes by optimizing immune responses and reducing excessive inflammation [52, 53]. Our findings indicate that Selnoflast has strong binding affinity for GATA3 and T-bet, suggesting potential effects on their interactions and supporting the synergistic role of the NLRP3/T-bet/GATA3 axis in orchestrating immune responses in UC.

The Truelove and Witts criteria, along with the Mayo and UCEIS scoring systems, are established scales for assessing UC severity and activity [3, 54, 55]. Their combined use enhances our understanding of cytokine profiles in UC, aiding in treatment target identification and evaluation of new therapies [55]. Our results indicated that NLRP3 inflammasome components (NLRP3, IL-1β, and IL-18) and cytokines associated with CD4 T cell subsets—Th2 (GATA-3, IL-4, IL-6), Th1 (T-bet, TNF-α), and Th17 (IL-17, IL-22)—were positively correlated with UC severity and activity. Further, NLRP3-P and IL-1β exhibited high diagnostic efficacy across all UC groups, underscoring the NLRP3 inflammasome's role in UC diagnosis. NLRP3, a critical regulatory factor of inflammation [56], shows increased NLRP3-P levels upon activation [57, 58]. IL-6 is a key mediator in driving Th2 responses, inhibiting Th1 differentiation, and promoting Th17 cell expansion, all of which exacerbate inflammation in UC [59, 60]. Elevated IL-6 levels, which correlate with increased disease activity, have high AUC values, making IL-6 a valuable biomarker for assessing UC severity, reinforcing the NLRP3/T-bet/GATA3 axis hypothesis since NLRP3 activation increases IL-6 production. Specifically, IL-6 downregulates T-bet expression, inhibiting the differentiation of Th1 cells towards IFN-γ producing cells, while upregulating GATA3 expression, thus favoring Th2 differentiation. Targeting IL-6 or its associated pathways may help restore Th1/Th2 balance, alleviating UC-related inflammatory responses. By suppressing T-bet, IL-6 hinders Th1 responses and upregulates GATA3 expression, thereby mechanistically demonstrating the feasibility of targeting this axis in combined therapy and might contribute to reducing inflammation more effectively and thereby enhance the therapeutic efficacy for UC [61, 62].

The study has several limitations. Firstly, the sample size remains constrained, which can be attributed to implementing stringent screening criteria and the preliminary phase of establishing our clinical database. Secondly, the design of this investigation limits our capacity to infer causality and temporal relationships. Future studies should employ more rigorous longitudinal cohort methodologies to attain a comprehensive understanding of the dynamic changes in CD4 T cell subsets and the NLRP3 inflammasome during UC progression. Thirdly, it is essential to consider the medication history of UC patients, as it may influence the interpretation of immune-related outcomes. As shown in Table 1, the only significant difference observed between patients with UC-LM and UC-S was the use of aminosalicylates. Aminosalicylates, such as mesalamine, are the first-line treatment for UC-LM, while patients with UC-S are less likely to use 5-ASA and instead rely more on corticosteroids, ISDs, and biologics for maintenance therapy [3, 63]. Different medication regimens can have varying effects on the immune system. For example, 5-ASA primarily acts as a local anti-inflammatory, reducing gut inflammation [64, 65], but its systemic immunosuppressive effects are minimal compared to corticosteroids and biologics, which can significantly modulate immune responses by altering T cell subsets and inflammasome activity [66–68]. Corticosteroids, for instance, may suppress both Th1 and Th17 cell activities [69], whereas ISDs and biologics can further modify the immune landscape by selectively targeting cytokine pathways such as TNF-α, IL-12, and IL-23 [70, 71]. To minimize potential confounding by drug effects, we accounted for this factor in our study design by classifying UC severity according to the Truelove and Witts criteria and assessing disease activity using the Mayo and UCEIS scores. Only patients with active disease (Mayo ≥ 3, UCEIS ≥ 1) were included, excluding those whose medication use might confound the results. However, we cannot entirely rule out the potential impact of certain medications on CD4 T cell subsets and NLRP3 inflammasome activity, which could introduce a minor bias in our results. Furthermore, the average age of the study cohort was 60 years or older, and gender differences could also exert confounding effects. Finally, our research does not include interventional studies aimed at directly evaluating the therapeutic potential of targeting the NLRP3/T-bet/GATA3 axis. Consequently, relevant multi-target combination clinical trials are necessary to assess the efficacy of modulating these pathways within UC therapy contexts.

## Conclusion

This study highlighted the intrinsic correlation between CD4 T cell subsets and the NLRP3 inflammasome, particularly elucidating that the synergistic potential of the NLRP3/T-bet/GATA3 axis may facilitate a combined immunomodulatory approach, offering a strategy worthy of exploration for multi-target combination therapy in UC and holding promise as valuable biomarkers for enhancing diagnostic accuracy and guiding treatment. However, the clinical efficacy of this combination therapy requires further validation and clarification through additional interventional studies.

## Supplementary Information

Supplementary Material 1.

## Data Availability

Data is provided within the manuscript or supplementary information files.
