# Supplementary material for: Correlation and diagnostic significance of CD4 T cell subsets and NLRP3 inflammasome in ulcerative colitis: the role of the NLRP3/T-bet/GATA3 axis
Source: BMC Gastroenterol. 2025 Jan 21;25:23. doi: 10.1186/s12876-025-03603-w (PMC11748810; doi:10.1186/s12876-025-03603-w)
Supplement: Supplementary file 1 — Supplementary Material 1. [file 12876_2025_3603_MOESM1_ESM.docx]

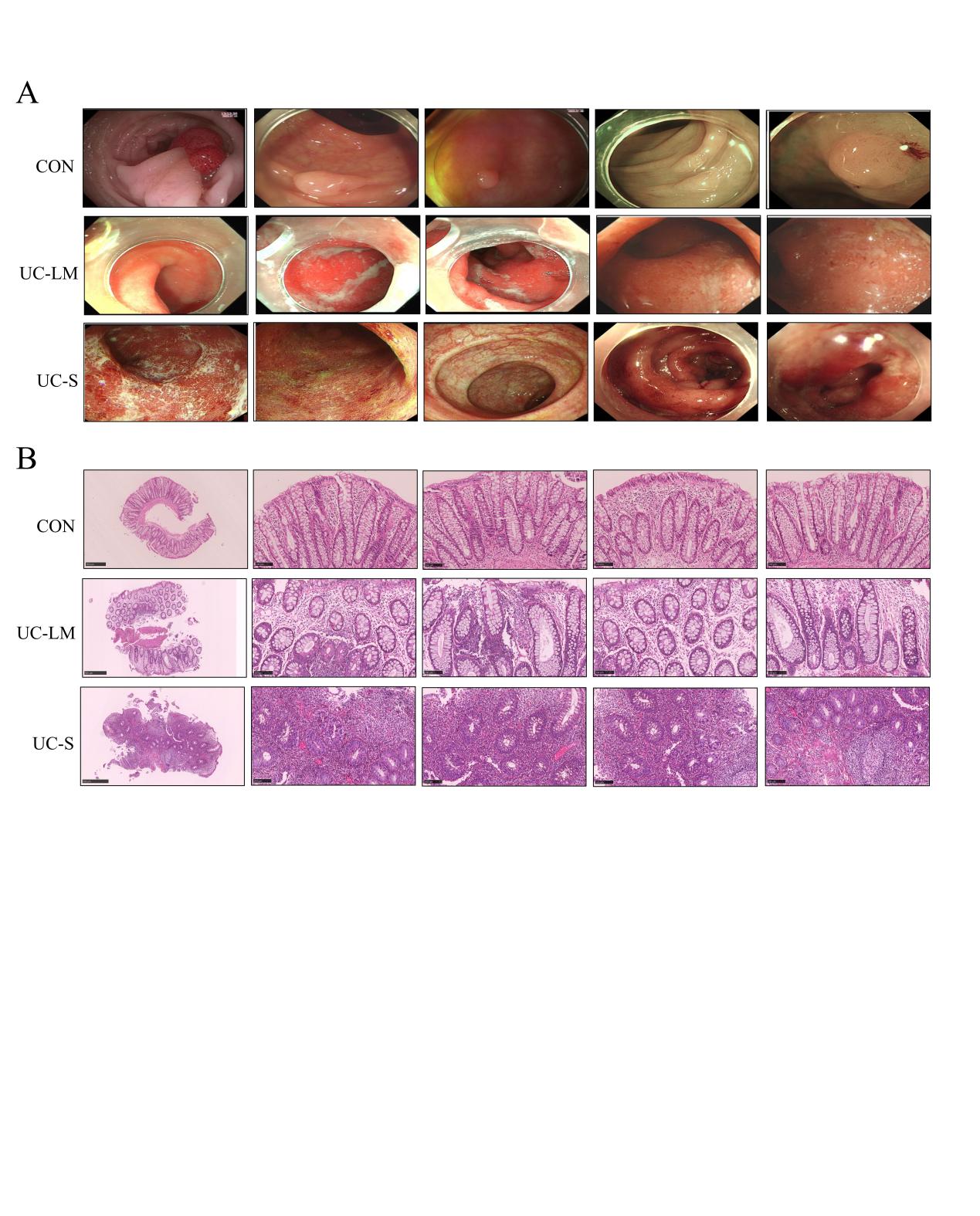
**Fig. S1** Colonoscopy and histopathological changes of UC patients and colon polyps controls. **A** Representative pictures of each group's colonoscopy. **B** Representative HE pathological stained colon sections (scale = 500 μm, multiple 40×; Scale = 100 μm, multiple 200×, select representative colon cross-section).

**Table** **S1** Diagnostic Accuracy Based on AUC Values

| **AUC Value** | **Diagnostic Accuracy** |
| --- | --- |
| Above 0.90 | High accuracy |
| 0.71 to 0.90 | Moderate accuracy |
| 0.50 to 0.70 | Low accuracy |
| Exactly 0.50 | No diagnostic value |
| Below 0.50 | Diagnostic inaccuracies |

**Table S2** Correlation between the indicators of both CD4 T cell subsets and NLRP3 inflammasome expressed as Spearman coefficient (*r*) and p-value (*p*).

|  |  | NLRP3  -F | IL-1β  -F | IL-18 | IFN-γ | TNF-α | IL-2 | IL-4 | IL-6 | IL-10 | IL-17 | TGF-  β1 | T-bet | GATA3 | RORγt | FOXP3 | NLRP3  -P | IL1β  -P | Caspase  -1 | IL-22 | IL-23 |
| --- | --- | --- | --- | --- | --- | --- | --- | --- | --- | --- | --- | --- | --- | --- | --- | --- | --- | --- | --- | --- | --- |
| NLRP3  -F | *r* | 1.000 | 0.769 | 0.371 | 0.093 | 0.550 | 0.246 | 0.311 | 0.499 | 0.162 | 0.405 | 0.132 | 0.524 | 0.499 | 0.453 | 0.195 | 0.573 | 0.700 | 0.279 | 0.378 | 0.358 |
|  | *p* | / | <0.001  (***) | 0.001  (**) | 0.439 | <0.001  (***) | 0.037  (*) | 0.008  (**) | <0.001  (***) | 0.175 | <0.001  (***) | 0.267 | <0.001  (***) | <0.001  (***) | <0.001  (***) | 0.102 | <0.001  (***) | <0.001  (***) | 0.018  (*) | 0.001  (**) | 0.002  (**) |
| IL-1β-F | *r* | 0.769 | 1.000 | 0.288 | 0.076 | 0.437 | 0.324 | 0.332 | 0.516 | 0.188 | 0.460 | 0.219 | 0.548 | 0.508 | 0.339 | 0.037 | 0.644 | 0.825 | 0.296 | 0.329 | 0.390 |
|  | *p* | <0.001  (***) | / | 0.014  (*) | 0.527 | <0.001  (***) | 0.006  (**) | 0.004  (**) | <0.001  (***) | 0.115 | <0.001  (***) | 0.064 | <0.001  (***) | <0.001  (***) | 0.004  (**) | 0.755 | <0.001  (***) | <0.001  (***) | 0.012  (*) | 0.005  (**) | 0.001  (**) |
| IL-18 | *r* | 0.371 | 0.288 | 1.000 | -0.074 | 0.319 | 0.050 | 0.110 | 0.154 | -0.015 | 0.465 | -0.176 | 0.299 | 0.537 | 0.218 | -0.031 | 0.368 | 0.285 | 0.142 | 0.188 | 0.155 |
|  | *p* | 0.001  (**) | 0.014  (*) | / | 0.535 | 0.006  (**) | 0.676 | 0.357 | 0.196 | 0.900 | <0.001  (***) | 0.140 | 0.011  (*) | <0.001  (***) | 0.066 | 0.796 | 0.001  (**) | 0.015  (*) | 0.234 | 0.113 | 0.194 |
| IFN-γ | *r* | 0.093 | 0.076 | -0.074 | 1.000 | -0.039 | 0.275 | -0.096 | 0.032 | 0.242 | 0.117 | 0.118 | -0.020 | 0.201 | 0.213 | 0.032 | 0.025 | -0.010 | 0.077 | 0.056 | -0.211 |
|  | *p* | 0.439 | 0.527 | 0.535 | / | 0.745 | 0.020  (*) | 0.422 | 0.793 | 0.041  (*) | 0.328 | 0.324 | 0.871 | 0.090 | 0.072 | 0.790 | 0.836 | 0.931 | 0.518 | 0.643 | 0.075 |
| TNF-α | *r* | 0.550 | 0.437 | 0.319 | -0.039 | 1.000 | 0.186 | 0.206 | 0.392 | 0.019 | 0.370 | 0.085 | 0.511 | 0.423 | 0.272 | 0.146 | 0.571 | 0.290 | 0.243 | 0.245 | 0.300 |
|  | *p* | <0.001  (***) | <0.001  (***) | 0.006  (**) | 0.745 | / | 0.117 | 0.082 | 0.001  (**) | 0.876 | 0.001  (**) | 0.479 | <0.001  (***) | <0.001  (***) | 0.021  (*) | 0.220 | <0.001  (***) | 0.013  (*) | 0.039  (*) | 0.038  (*) | 0.010  (*) |
| IL-2 | *r* | 0.246 | 0.324 | 0.050 | 0.275 | 0.186 | 1.000 | 0.233 | 0.227 | 0.200 | 0.420 | -0.008 | -0.001 | 0.035 | -0.030 | 0.251 | 0.098 | 0.419 | -0.047 | 0.050 | 0.219 |
|  | *p* | 0.037  (*) | 0.006  (**) | 0.676 | 0.020  (*) | 0.117 | / | 0.049  (*) | 0.055 | 0.091 | <0.001  (***) | 0.947 | 0.996 | 0.773 | 0.804 | 0.033  (*) | 0.414 | <0.001  (***) | 0.696 | 0.675 | 0.064 |
| IL-4 | *r* | 0.311 | 0.332 | 0.110 | -0.096 | 0.206 | 0.233 | 1.000 | 0.432 | -0.089 | 0.259 | -0.094 | 0.377 | 0.246 | 0.286 | -0.118 | 0.326 | 0.365 | 0.211 | 0.439 | 0.350 |
|  | *p* | 0.008  (**) | 0.004  (**) | 0.357 | 0.422 | 0.082 | 0.049  (*) | / | <0.001  (***) | 0.456 | 0.028  (*) | 0.430 | 0.001  (**) | 0.037  (*) | 0.015  (*) | 0.322 | 0.005  (**) | 0.002  (**) | 0.075 | <0.001  (***) | 0.003  (**) |
| IL-6 | *r* | 0.499 | 0.516 | 0.154 | 0.032 | 0.392 | 0.227 | 0.432 | 1.000 | 0.219 | 0.474 | 0.213 | 0.566 | 0.452 | 0.498 | 0.032 | 0.563 | 0.470 | 0.435 | 0.626 | 0.502 |
|  | *p* | <0.001  (***) | <0.001  (***) | 0.196 | 0.793 | 0.001  (**) | 0.055 | <0.001  (***) | / | 0.065 | <0.001  (***) | 0.073 | <0.001  (***) | <0.001  (***) | <0.001  (***) | 0.787 | <0.001  (***) | <0.001  (***) | <0.001  (***) | <0.001  (***) | <0.001  (***) |
| IL-10 | *r* | 0.162 | 0.188 | -0.015 | 0.242 | 0.019 | 0.200 | -0.089 | 0.219 | 1.000 | 0.171 | 0.731 | -0.059 | 0.250 | 0.331 | 0.293 | 0.248 | 0.142 | 0.186 | -0.167 | 0.138 |
|  | *p* | 0.175 | 0.115 | 0.900 | 0.041  (*) | 0.876 | 0.091 | 0.456 | 0.065 | / | 0.151 | <0.001  (***) | 0.623 | 0.034  (*) | 0.004  (**) | 0.013  (*) | 0.036  (*) | 0.234 | 0.118 | 0.160 | 0.246 |
| IL-17 | *r* | 0.405 | 0.460 | 0.465 | 0.117 | 0.370 | 0.420 | 0.259 | 0.474 | 0.171 | 1.000 | -0.010 | 0.322 | 0.406 | 0.151 | 0.119 | 0.436 | 0.528 | 0.126 | 0.211 | 0.377 |
|  | *p* | <0.001  (***) | <0.001  (***) | <0.001  (***) | 0.328 | 0.001  (**) | <0.001  (***) | 0.028  (*) | <0.001  (***) | 0.151 | / | 0.931 | 0.006  (**) | <0.001  (***) | 0.206 | 0.320 | <0.001  (***) | <0.001  (***) | 0.292 | 0.075 | 0.001  (**) |
| TGF-β1 | *r* | 0.132 | 0.219 | -0.176 | 0.118 | 0.085 | -0.008 | -0.094 | 0.213 | 0.731 | -0.010 | 1.000 | 0.061 | 0.110 | 0.322 | 0.221 | 0.298 | 0.131 | 0.260 | -0.114 | 0.079 |
|  | *p* | 0.267 | 0.064 | 0.140 | 0.324 | 0.479 | 0.947 | 0.430 | 0.073 | <0.001  (***) | 0.931 | / | 0.608 | 0.360 | 0.006  (**) | 0.062 | 0.011  (*) | 0.273 | 0.028  (*) | 0.339 | 0.511 |
| T-bet | *r* | 0.524 | 0.548 | 0.299 | -0.020 | 0.511 | -0.001 | 0.377 | 0.566 | -0.059 | 0.322 | 0.061 | 1.000 | 0.705 | 0.614 | 0.030 | 0.765 | 0.351 | 0.370 | 0.428 | 0.237 |
|  | *p* | <0.001  (***) | <0.001  (***) | 0.011  (*) | 0.871 | <0.001  (***) | 0.996 | 0.001  (**) | <0.001  (***) | 0.623 | 0.006  (**) | 0.608 | / | <0.001  (***) | <0.001  (***) | 0.805 | <0.001  (***) | 0.003  (**) | 0.001  (**) | <0.001  (***) | 0.045  (*) |
| GATA3 | *r* | 0.499 | 0.508 | 0.537 | 0.201 | 0.423 | 0.035 | 0.246 | 0.452 | 0.250 | 0.406 | 0.110 | 0.705 | 1.000 | 0.666 | -0.019 | 0.729 | 0.291 | 0.476 | 0.322 | 0.167 |
|  | *p* | <0.001  (***) | <0.001  (***) | <0.001  (***) | 0.090 | <0.001  (***) | 0.773 | 0.037  (*) | <0.001  (***) | 0.034  (*) | <0.001  (***) | 0.360 | <0.001  (***) | / | <0.001  (***) | 0.872 | <0.001  (***) | 0.013  (*) | <0.001  (***) | 0.006  (**) | 0.162 |
| RORγt | *r* | 0.453 | 0.339 | 0.218 | 0.213 | 0.272 | -0.030 | 0.286 | .498 | 0.331 | 0.151 | 0.322 | 0.614 | 0.666 | 1.000 | 0.107 | 0.605 | 0.138 | 0.486 | 0.357 | 0.129 |
|  | *p* | <0.001  (***) | 0.004  (**) | 0.066 | 0.072 | 0.021  (*) | 0.804 | 0.015  (*) | <0.001  (***) | 0.004  (**) | 0.206 | 0.006  (**) | <0.001  (***) | <0.001  (***) | / | 0.372 | <0.001  (***) | 0.249 | <0.001  (***) | 0.002  (**) | 0.279 |
| FOXP3 | *r* | 0.195 | 0.037 | -0.031 | 0.032 | 0.146 | 0.251 | -0.118 | 0.032 | 0.293 | 0.119 | 0.221 | 0.030 | -0.019 | 0.107 | 1.000 | 0.222 | 0.061 | 0.003 | -0.055 | 0.234 |
|  | *p* | 0.102 | 0.755 | 0.796 | 0.790 | 0.220 | 0.033  (*) | 0.322 | 0.787 | 0.013  (*) | 0.320 | 0.062 | 0.805 | 0.872 | 0.372 | / | 0.061 | 0.612 | 0.982 | 0.646 | 0.048  (*) |
| NLRP3  -P | *r* | 0.573 | 0.644 | 0.368 | 0.025 | 0.571 | 0.098 | 0.326 | 0.563 | 0.248 | 0.436 | 0.298 | 0.765 | 0.729 | 0.605 | 0.222 | 1.000 | 0.439 | 0.467 | 0.282 | 0.411 |
|  | *p* | <0.001  (***) | <0.001  (***) | 0.001  (**) | 0.836 | <0.001  (***) | 0.414 | 0.005  (**) | <0.001  (***) | 0.036  (*) | <0.001  (***) | 0.011  (*) | <0.001  (***) | <0.001  (***) | <0.001  (***) | 0.061 | / | <0.001  (***) | <0.001  (***) | 0.016  (*) | <0.001  (***) |
| IL-1β-P | *r* | 0.700 | 0.825 | 0.285 | -0.010 | 0.290 | 0.419 | 0.365 | 0.470 | 0.142 | 0.528 | 0.131 | 0.351 | 0.291 | 0.138 | 0.061 | 0.439 | 1.000 | 0.106 | 0.259 | 0.294 |
|  | *p* | <0.001  (***) | <0.001  (***) | 0.015  (*) | 0.931 | 0.013  (*) | <0.001  (***) | 0.002  (**) | <0.001  (***) | 0.234 | <0.001  (***) | 0.273 | 0.003  (**) | 0.013  (*) | 0.249 | 0.612 | <0.001  (***) | / | 0.376 | 0.028  (*) | 0.012  (*) |
| Caspase  -1 | *r* | 0.279 | 0.296 | 0.142 | 0.077 | 0.243 | -0.047 | 0.211 | 0.435 | 0.186 | 0.126 | 0.260 | 0.370 | 0.476 | 0.486 | 0.003 | 0.467 | 0.106 | 1.000 | 0.337 | 0.125 |
|  | *p* | 0.018  (*) | 0.012  (*) | 0.234 | 0.518 | 0.039  (*) | 0.696 | 0.075 | <0.001  (***) | 0.118 | 0.292 | 0.028  (*) | 0.001  (**) | <0.001  (***) | <0.001  (***) | 0.982 | <0.001  (***) | 0.376 | / | 0.004  (**) | 0.297 |
| IL-22 | *r* | 0.378 | 0.329 | 0.188 | 0.056 | 0.245 | 0.050 | 0.439 | 0.626 | -0.167 | 0.211 | -0.114 | 0.428 | 0.322 | 0.357 | -0.055 | 0.282 | 0.259 | 0.337 | 1.000 | 0.225 |
|  | *p* | 0.001  (**) | 0.005  (**) | 0.113 | 0.643 | 0.038  (*) | 0.675 | <0.001  (***) | <0.001  (***) | 0.160 | 0.075 | 0.339 | <0.001  (***) | 0.006  (**) | 0.002  (**) | 0.646 | 0.016  (*) | 0.028  (*) | 0.004  (**) | / | 0.057 |
| IL-23 | *r* | 0.358 | 0.390 | 0.155 | -0.211 | 0.300 | 0.219 | 0.350 | 0.502 | 0.138 | 0.377 | 0.079 | 0.237 | 0.167 | 0.129 | 0.234 | 0.411 | 0.294 | 0.125 | 0.225 | 1.000 |
|  | *p* | 0.002  (**) | 0.001  (**) | 0.194 | 0.075 | 0.010  (*) | 0.064 | 0.003  (**) | <0.001  (***) | 0.246 | 0.001  (**) | 0.511 | 0.045  (*) | 0.162 | 0.279 | 0.048  (*) | <0.001  (***) | 0.012  (*) | 0.297 | 0.057 | / |

**Table S3** The indicators of both CD4 T cell subsets and NLRP3 inflammasome correlation with the severity of UC, Mayo score, and UCEIS expressed as Spearman coefficient (r) and p-value (*p*).

| Indicators | Correlation | Severity of UC | Mayo score | UCEIS |
| --- | --- | --- | --- | --- |
| NLRP3-F | *r* | 0.672 | 0.451 | 0.524 |
|  | *p* | <0.001 (***) | 0.003 (**) | <0.001 (***) |
| IL-1β-F | *r* | 0.788 | 0.343 | 0.359 |
|  | *p* | <0.001 (***) | 0.026 (*) | 0.020 (*) |
| IL-18 | *r* | 0.484 | 0.618 | 0.690 |
|  | *p* | <0.001 (***) | <0.001 (***) | <0.001 (***) |
| IFN-γ | *r* | -0.047 | -0.052 | 0.033 |
|  | *p* | 0.698 | 0.744 | 0.838 |
| TNF-α | *r* | 0.601 | 0.514 | 0.378 |
|  | *p* | <0.001 (***) | 0.001 (**) | 0.014 (**) |
| IL-2 | *r* | 0.346 | 0.076 | 0.141 |
|  | *p* | 0.006 (**) | 0.633 | 0.374 |
| IL-4 | *r* | 0.322 | 0.402 | 0.476 |
|  | *p* | 0.003 (**) | 0.008 (**) | 0.001 (**) |
| IL-6 | *r* | 0.653 | 0.395 | 0.407 |
|  | *p* | <0.001 (***) | 0.010 (*) | 0.007 (**) |
| IL-10 | *r* | 0.198 | -0.508 | -0.542 |
|  | *p* | 0.096 | 0.001 (**) | <0.001 (***) |
| IL-17 | *r* | 0.643 | 0.480 | 0.469 |
|  | *p* | <0.001 (***) | 0.001 (**) | 0.002 (**) |
| TGF-β1 | *r* | 0.158 | -0.700 | -0.674 |
|  | *p* | 0.184 | <0.001 (***) | <0.001 (***) |
| T-bet | *r* | 0.561 | 0.645 | 0.594 |
|  | *p* | <0.001 (***) | <0.001 (***) | <0.001 (***) |
| GATA3 | *r* | 0.570 | 0.836 | 0.689 |
|  | *p* | <0.001 (***) | <0.001 (***) | <0.001 (***) |
| RORγt | *r* | 0.338 | 0.357 | 0.296 |
|  | *p* | 0.004 (**) | 0.020 (*) | 0.057 |
| FOXP3 | *r* | 0.144 | -0.129 | -0.282 |
|  | *p* | 0.228 | 0.417 | 0.071 |
| NLRP3-P | *r* | 0.694 | 0.625 | 0.545 |
|  | *p* | <0.001 (***) | <0.001 (***) | <0.001 (***) |
| IL-1β-P | *r* | 0.664 | 0.343 | 0.359 |
|  | *p* | <0.001 (***) | <0.026 (*) | <0.020 (*) |
| Caspase-1 | *r* | 0.328 | 0.097 | 0.111 |
|  | *p* | 0.005 (**) | 0.541 | 0.485 |
| IL-22 | *r* | 0.346 | 0.535 | 0.621 |
|  | *p* | 0.003 (**) | <0.001 (***) | <0.001 (***) |
| IL-23 | *r* | 0.594 | 0.263 | 0.360 |
|  | *p* | <0.001 (***) | 0.092 | 0.019 (*) |

**Table S4** Area under the ROC curve, cut-off values, sensitivity and specificity of core indicators of both CD4 T cell subsets and NLRP3 inflammasome in assessment of UC-S patients.

| Core indicators | AUC | 95%Cl | *P* value | Cut-off value | Sensitivity (%) | Specificity (%) |
| --- | --- | --- | --- | --- | --- | --- |
| IL-1β-F | 0.9105 | 0.8410-0.9890 | <0.0001 | 11.30 pg/ml | 83.33% | 73.33% |
| IL-17 | 0.9000 | 0.8157-0.9843 | <0.0001 | 26.31 pg/ml | 75.00% | 93.33% |
| NLRP3-P | 0.8983 | 0.7985-0.9982 | <0.0001 | 0.3435 | 75.00% | 100.00% |
| TNF-α | 0.8950 | 0.7637-1.000 | <0.0001 | 3.240 pg/ml | 85.00% | 100.00% |
| IL-6 | 0.8917 | 0.7973-0.9860 | <0.0001 | 18.95 pg/ml | 80.00% | 93.33% |
| GATA3 | 0.8650 | 0.7530-0.9770 | <0.0001 | 0.3076 | 70.00% | 96.67% |
| NLRP3-F | 0.8583 | 0.7335-0.9831 | <0.0001 | 34.54 pg/ml | 75.00% | 96.67% |
| IL-18 | 0.8533 | 0.7096-0.9971 | <0.0001 | 199.1 pg/ml | 80.00% | 100.00% |
| T-bet | 0.8450 | 0.7082-0.9818 | <0.0001 | 0.2737 pg/ml | 75.00% | 93.33% |
| IL-4 | 0.7967 | 0.6584-0.9350 | 0.0004 | 0.4350 pg/ml | 85.00% | 76.67% |
| IL-1β-P | 0.7950 | 0.6655-0.9245 | 0.0005 | 0.2801 | 75.00% | 76.67% |
| IL-22 | 0.7908 | 0.6522-0.9294 | 0.0005 | 16.95 pg/ml | 80.00% | 76.67% |

**Table S5** Supplementation of the results of differentially expressed indicators of Ulcerative colitis (UC) patients and Colon polyp controls (CON)

| Differentially expressed indicators | CON group  (n = 30) | UC-LM group  (n = 22) | UC-S group (n = 20) | *P* value^a^ | *P* value^b^ | *P* value^c^ | *P* value^d^ |
| --- | --- | --- | --- | --- | --- | --- | --- |
| CD4 T cell subsets  (the transcriptional proteins)   1. bet   GATA3  RORγt  FOXP3  (the related cytokines)  TNF-α  IFN-γ  IL-2  IL-4  IL-6  IL-10  IL-17  IL-22  IL-23  TGF-β1 | 0.2591  (0.2513-0.2671)  0.2814 ±  0.003095  0.3238 ±  0.003349  0.2926 ±  0.003185  2.389  (1.980-2.789)  0.3377  (0.1039- 1.062)  2.572  (1.356-9.239)  0.2287  (0.1229-0.4512)  0.7193  (2.864-47.43)  3.187  (2.337-3.712)  1.553  (0.8000-14.12)  11.40  (7.872-17.17)  65.44  (56.66-84.09)  2215 ± 136.5 | 0.2686  (0.2607-0.2769)  0.3007 ±  0.003430  0.3452 ±  0.004542  0.3091 ±  0.002236  2.789 (2.382-5.071 )  0.8228 (0.1538-4.085) 4.679 (2.646-6.524)  0.1537  (0.09678-1.102)  11.13  (4.995-19.78)  20.67  (18.73-22.94)  10.86  (4.622-23.36)  10.61  (5.77-15.60)  94.84  (79.26-125.6)  5274 ± 116.5 | 0.2848 (0.2712-0.2910)  0.3231 ±  0.007279  0.3427 ±  0.0063792  0.2907 ±  0.002625  8.937 (4.771-10.00)  0.4153 (0.0865-1.015)  11.39 (2.969-19.07)  1.152  (0.5043-1.483)  46.55  (26.51-96.85)  3.021  (2.515-4.415)  42.43  (14.32-87.11)  28.35  (17.74-32.23)  147.5  (128.9-229.4)  2300 ± 134.7 | 0.0315  0.0077  0.0032  0.0002  0.0487  0.7892  0.4655  >0.9999  0.0146  <0.0001  0.0382  >0.9999  0.0373  <0.0001 | <0.0001  <0.0001  0.0131  0.8905  <0.0001  >0.9999  0.0221  0.0039  <0.0001  >0.9999  <0.0001  0.0024  <0.0001  0.8936 | 0.0987  0.0047  0.9294  0.0002  0.0368  >0.9999  0.7122  0.0031  0.0314  <0.0001  0.0186  0.0004  0.0499  <0.0001 | <0.0001  <0.0001  0.0015  <0.0001  <0.0001  0.5026  0.0258  0.0012  <0.0001  <0.0001  <0.0001  0.0002  <0.0001  <0.0001 |
| NLRP3 inflammasome  (the core proteins)  NLRP3-P  Caspase-1  IL-1β-P  (the related factors)  NLRP3-F  IL-1β  IL-18 | 0.3097 ±  0.003522  0.2761 ±  0.001685  0.2763  (0.2684-0.2804)  0.7303  (0.3278- 9.505)  1.806  (0.4313- 14.52)  63.07  (21.37-100.4) | 0.3401 ±  0.003560  0.2922 ±  0.004991  0.2998  (0.2911-0.3097)  12.56  (6.063-22.11)  15.58  (8.153-18.00)  67.08  (18.43-154.2 ) | 0.3737 ±  0.01853  0.2970 ±  0.007563  0.2861  (0.2788-0.3989)  193.5  (25.70-281.4)  26.67 (18.30-37.25)  347.0 (281.7-404.9) | 0.0136  0.0363  <0.0001  0.0074  0.0303  0.9037 | <0.0001  0.0060  0.0004  <0.0001  <0.0001  <0.0001 | 0.0243  0.7736  0.3820  0.0392  0.0104  0.0065 | <0.0001  0.0040  <0.0001  <0.0001  <0.0001  <0.0001 |

*UC* ulcerative colitis, *UC-LM* mild to moderate ulcerative colitis, *UC-S* severe UC, *CON* colon polyp controls.

^a^ comparing CON group and UC-LM group

^b^ comparing CON group group and UC-S group

^c^ comparing UC-LM group and UC-S group

^d^ comparing CON group, UC-LM group and UC-S group
